# Evaluation of Telehealth Visit Attendance After Implementation of a Patient Navigator Program

**DOI:** 10.1001/jamanetworkopen.2022.45615

**Published:** 2022-12-08

**Authors:** Oren J Mechanic, Emma M. Lee, Heidi M. Sheehan, Tenzin Dechen, Ashley L. O’Donoghue, Timothy S. Anderson, Catherine Annas, Leanne B. Harvey, Allison A. Perkins, Michael A. Severo, Jennifer P. Stevens, Alexa B. Kimball

**Affiliations:** 1 Harvard Medical Faculty Physicians, Beth Israel Deaconess Medical Center, Boston, Massachusetts; 2 Center for Healthcare Delivery Science, Beth Israel Deaconess Medical Center, Boston, Massachusetts; 3 Division of General Medicine, Beth Israel Deaconess Medical Center, Boston, Massachusetts; 4 Harvard Medical School, Boston, Massachusetts

## Abstract

**Importance:**

The dramatic rise in use of telehealth accelerated by COVID-19 created new telehealth-specific challenges as patients and clinicians adapted to technical aspects of video visits.

**Objective:**

To evaluate a telehealth patient navigator pilot program to assist patients in overcoming barriers to video visit access.

**Design, Setting, and Participants:**

This quality improvement study investigated visit attendance outcomes among those who received navigator outreach (intervention group) compared with those who did not (comparator group) at 2 US academic primary care clinics during a 12-week study period from April to July 2021. Eligible participants had a scheduled video visit without previous successful telehealth visits.

**Interventions:**

The navigator contacted patients with next-day scheduled video appointments by phone to offer technical assistance and answer questions on accessing the appointment.

**Main Outcomes and Measures:**

The primary outcome was appointment attendance following the intervention. Return on investment (ROI) accounting for increased clinic adherence and costs of implementation was examined as a secondary outcome.

**Results:**

A total 4066 patients had video appointments scheduled (2553 [62.8%] women; median [IQR] age: intervention, 55 years [38-66 years] vs comparator, 52 years [36-66 years]; *P* = .02). Patients who received the navigator intervention had significantly increased odds of attending their appointments (odds ratio, 2.0; 95% CI, 1.6-2.6) when compared with the comparator group, with an absolute increase of 9% in appointment attendance for the navigator group (949 of 1035 patients [91.6%] vs 2511 of 3031 patients [82.8%]). The program’s ROI was $11 387 over the 12-week period.

**Conclusions and Relevance:**

In this quality improvement study, we found that a telehealth navigator program was associated with significant improvement in video visit adherence with a net financial gain. Our findings have relevance for efforts to reduce barriers to telehealth-based health care and increase equity.

## Key Points

**Question:**

Was the introduction of patient navigator prior to telehealth video appointments associated with improved attendance to telemedicine visits?

**Findings:**

In this quality improvement study conducted at a single US health center with 4066 patients, there was an absolute increase of 9% in appointment attendance with the use of a patient navigator (92% vs 83%). The program had a positive return on investment of $11 387 over the 12-week period, accounting for program costs and average reimbursement.

**Meaning:**

The findings of this study suggest that adding a patient navigator to episodic telehealth visits may increase visit attendance and provide a net financial return.

## Teaser

This quality improvement study evaluates the implementation of a patient navigator pilot program to increase attendance at telehealth visits.

## Introduction

Since the onset of the COVID-19 pandemic, synchronous telemedicine visits, whether by phone or video, have increased dramatically. Physicians at Harvard Medical Faculty Physicians at Beth Israel Deaconess Medical Center, a major academic teaching hospital in Boston, Massachusetts, rapidly adopted telehealth but reported technical challenges during video visits and sometimes converted video visits to telephone.^1^

Patient outreach has been suggested to facilitate successful video visits,^2^ and during the launch of over 500 000 ambulatory telehealth visits at our medical center since the onset of the COVID-19 pandemic, we implemented a pilot program to address the transition from telephone to video visits. Patient navigators are typically identified as staff that guide patients in coordination of care, appointment adherence, and discharge planning.^3,4^ Our objective was to evaluate the implementation of a novel patient navigator to reduce barriers to successful video visit use. Prior studies of patient navigators have suggested that the cost-effectiveness of such programs are uncertain.^5^ While measuring the outcomes of this pilot, we also calculated the cost and return on investment (ROI) of the pilot program.

## Methods

This study was reviewed by the Beth Israel Deaconess Medical Center institutional review board and was deemed exempt from informed consent in accordance with the Common Rule. The study follows the Standards for Quality Improvement Reporting Excellence (SQUIRE) reporting guideline.

### Program Implementation & Data Collection

A trained Telehealth Patient Navigator (hereafter *navigator*) was hired and trained in-person and virtually over 6 to 8 hours to familiarize with video visit workflow, data entry, and the electronic health record (EHR). Patients were contacted 1 day prior to video appointments with the goal of improving access to care by reducing technological barriers prior to joining the video visit to those who were new to using the modality. The navigator attempted to reach patients with morning and afternoon appointments in equal numbers. A navigator script that covered the steps required for the patient to connect to their visit and a set of frequently asked questions were given to the navigator. Thirty-minute check-ins were done with the supervising team over the course of 1 to 2 weeks and as needed thereafter. Patients received either phone-based outreach from the navigator (intervention group), or usual communication (comparator group). Usual communication included typical appointment reminders, such as phone calls and texts sent by the clinics, and those in the navigator group received such intervention as well. The primary care and gerontology clinics were selected as pilots to address a heterogeneous and presumed at-risk population. Over the course of a day, the navigator typically reached out to 27 patients and successfully contacted approximately 22 patients (up to 44 per day), depending on the daily bandwidth of the navigator. All patients in the gerontology clinic were attempted daily, and we asked that the navigator call an equal number of morning and afternoon primary care clinic patients.

The navigator was hired and trained the week of April 12, 2021, and began calls independently on April 19, 2021. Operationally, the pilot concluded 12 weeks later, and we analyzed the initial results. A total 1035 of 4068 patient visits were reached out to by the navigator—2 had no known outcome (Figure). The navigator logged information following each of their outreach attempts, including whether the patient was successfully reached, the services administered, approximate length of time spent on the call, and patient readiness confidence as reported by the patient. Final visit type and appointment attendance were collected from the clinician’s final visit method (ie, phone, video, in-person, did not keep, or cancel) in the scheduling system warehouse.

**Figure.**
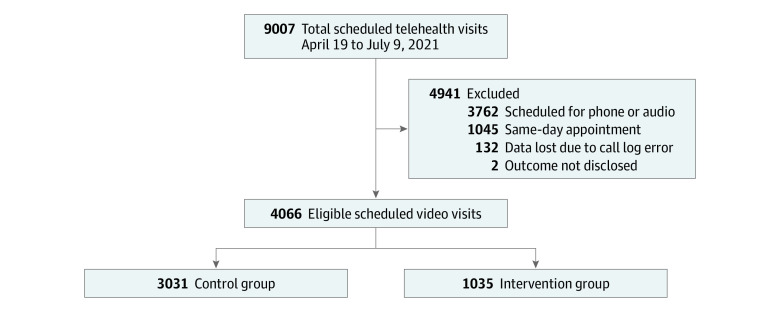
Study Group Selection Flowchart

### Statistical Analysis

We compared the proportion of appointments kept between the intervention group and comparator group using a χ^2^ test. Using multivariate logistic regression, we determined the adjusted odds ratios of appointment attendance (attended vs not attended) in the intervention vs comparator group. Other covariates included age, sex, race, and language as identified by patients in the EHR. Race was self-reported and was reported as appeared in the patient’s record. Race was reported in the study to elucidate if access to care and program effectiveness varied by race. Continuous variables were compared using 2-sample *t* tests and categorical variables using χ^2^ tests. All patients that the navigator attempted to reach (even if unsuccessful) were attributed to the intervention group as intention to treat.

### Program Value

Benefits were calculated as (1) the difference in video visit success rate between the intervention and comparator groups, plus (2) the difference in the canceled or no-show (cancellation) rate between the comparator and intervention groups minus the cost of the navigator salary. Each visit was estimated conservatively as 1.5 relative value units (RVUs) of the approximate average Medicare RVU, $40 reimbursement per RVU, and no facility revenue. We performed sensitivity testing of our visit reimbursement assumptions by varying average RVU, RVU reimbursement (utilizing an institutional payer-mix of $65 per RVU), and facility charges to adjust for possible commercial changes. The pilot cost was factored in at $17 878 for navigator salary plus fringe for 12 weeks.

## Results

In our evaluation of program effectiveness, we had 4068 adults with video appointments at an academic hospital’s primary care and gerontology clinics scheduled between April 19, 2021, and July 9, 2021. Across both intervention and comparator groups, 2553 participants (62.8%) were women (navigator, 654 women [63.2%] vs comparator, 1899 women [62.7%]; *P* = .79) (Table 1). Median (IQR) age was 3 years older in the intervention group vs the comparator (55 [38-66] years vs 52 [36-66] years; *P* = .02). The majority of patients in each group were from the primary care clinic (intervention, 993 [95.9%]; comparator, 2948 [97.3%]) and the remainder from the smaller gerontology clinic (42 [4.1%] and 83 [2.7%], respectively). A total of 4066 had a known visit outcome (with 2 lost to follow up) and were included in the analyses for program effectiveness. Of that sample, the intervention group was comprised of 1035 (25.5%) patients who were attempted to be reached or successfully reached by the navigator. The comparator group comprised 3033 patients (74.6%) within the same clinics during the same 12-week period who the navigator did not attempt to reach.

**Table 1.** Study Population Demographic Characteristics, by Navigator Flag Status (N = 4066)

Characteristics	Visits, No. (%)		*P* value
	Navigator intervention (n = 1035)	Comparator (n = 3031)	
Age, median (IQR), y	55 (38-66)	52 (36-66)	.02^a^
Sex			
Men	381 (36.8)	1132 (37.3)	.79^b^
Women	654 (63.2)	1899 (62.7)	
Race			
African American and Black	220 (21.3)	709 (23.4)	.003^b^
American Indian and Alaska Native, Native Hawaiian and Pacific Islander	7 (0.7)	8 (0)	
Asian	49 (4.7)	181 (6.0)	
White	662 (64.0)	1755 (57.9)	
Other^c^	48 (4.6)	287 (9.5)	
Unknown^d^	49 (4.7)	191 (6.3)	
Clinic			
Primary care	993 (95.9)	2948 (97.3)	.04^b^
Gerontology	42 (4.1)	83 (2.7)	

^a^ Denotes 2-sample *t* test.

^b^ Denotes χ^2^ test.

^c^ Other race was an option available in the electronic health record.

^d^ Unknown included blank, unknown (an option in the electronic health record), and declined to answer.

Unadjusted analysis showed that 949 of 1035 patients (91.6%) from the intervention group successfully attended their appointment compared with 2511 of 3031 (82.8%) in the comparator group (*P* < .001) (Table 2). Sixty patients (5.8%) in the intervention group patients canceled their appointment compared with 279 (9.2%) in the comparator group (*P* < .001). Twenty-six patients (2.5%) of the intervention group missed the appointment compared with 241 (8%) in the comparator group (*P* < .001).

**Table 2.** Video Visit Success by Pilot Group

Visit status	Patients, No. (%)		*P* value^a^
	Navigator intervention (n = 1035)	Comparator (n = 3031)^b^	
Kept (attended a visit)	949 (91.6)	2511 (82.8)	<.001
Video	836 (80.8)	1805 (59.6)	
Converted to telephone	111 (10.7)	689 (22.7)	
Converted to in person	2 (0.2)	17 (0.6)	
Appointment canceled	60 (5.8)	279 (9.2)	
Did not keep (no show)	26 (2.5)	241 (8.0)	

^a^ *P* value denotes χ^2^ test with 3 categories: kept, appointment canceled, and did not keep. The additional detailed modality of visits which were kept (ie, video, telephone, and in-person) are provided here to support calculations for return on investment.

^b^ Comparator group data source only has visits to be dispositioned as of day of the visit and does not have visits moved prior to visit day.

Patients in the intervention group had an adjusted odds ratio (aOR) of 2.0 (95% CI, 1.6-2.6) of appointment attendance compared with the comparator group (eTable in the Supplement). Patients with morning appointments were more likely to attend their appointment (aOR, 1.2; 95% CI, 1.0-1.5) relative to afternoon appointments (eFigure in the Supplement). There was no statistically significant difference in appointment attendance for patients over 65 years old (aOR, 1.2; 95% CI, 0.9-1.5) relative to patients aged 45 to 65 years (eFigure in the Supplement). Black patients were less likely to attend (aOR, 0.5; 95% CI, 0.4-0.6) relative to White patients. Other patients who were less likely to attend were patients who spoke languages other than English (aOR, 0.6; 95% CI, 0.5-0.9) or without a language documented (aOR, 0.3; 95% CI, 0.1-0.9) compared with English speakers.

Of the 1035 patients in the intervention group, 300 (29.0%) were projected to have had unsuccessful video visits without the navigator based on the intervention vs comparator group success and cancellation rate differences. Specifically, in the primary care and gerontology clinic, the intervention group had a 21% absolute increase in the video visit success rate compared with the comparator group (81% vs 60%; *P* < .001), resulting in 217 additional video visits. Additionally, the missed appointment and cancellation rate was 8 percentage points lower in the intervention group (9%) vs the comparator group (17%), resulting in 83 additional visits.

### Program Value

At $65 per RVU reimbursement, the 300 additional visits yielded $29 265 (Table 3). Accounting for the quarterly navigator salary of $17 878, the estimated ROI of the Telehealth Navigator Program was actualized at $11 387 over 12 weeks for the institutional payer-mix (Table 4). For the approximate Medicare average of $40 per RVU, the ROI was $131 over a 12-week period. Reimbursement assumption sensitivity analysis showed ROI remained neutral at $39 per RVU without a facility fee, and with payment parity between audio-only and video telehealth reimbursement.

**Table 3.** Navigator Intervention Pilot Program Return on Investment (ROI)

Characteristic	Navigator intervention, No.	Navigator intervention rate, %^a^	Comparator group rate, %^a^	Navigator visit difference, No.^b^	Reimbursement estimate associated with visit difference, $^c^
**Benefits**					
Additional video visits completed	1035	81	60	217	21 192
Fewer no show and canceled video visits	1035	9	17	83	8073
Fewer video visits switched to telephone visits	1035	11	23	123	0^d^
Saved clinician technical troubleshooting time	NA	NA	NA	NA	Not measured
Improved patient experience with video platform	NA	NA	NA	NA	Not measured
Total benefits	NA	NA	NA	NA	29 265
**Costs**					
Navigator grade 7 salary and fringe 12 weeks	NA	NA	NA	NA	17 878
Training, management, phone script development, license	NA	NA	NA	NA	Not measured
Total costs	NA	NA	NA	NA	17 878
ROI^e^	NA	NA	NA	NA	11 387

Abbreviation: NA, not applicable.

^a^ Navigator intervention and comparator group rates are weighted averages of the Gerontology and Primary Medicine Clinics.

^b^ Navigator visit difference was determined as the absolute difference of the formula *Navigator intervention* × (*Navigator intervention rate* − *Comparator rate*).

^c^ $65 per revenue value unit (RVU) and 1.5 RVU per visit assumed in our Institutional Specific baseline model.

^d^ No revenue reimbursement difference between video and telephone visits assumed in baseline model.

^e^ Calculated as the total difference of benefits and costs.

**Table 4.** Return on Investment Model Sensitivity Analysis Calculations Over 12-Week Period

Analysis	RVUs per visit	Cost per RVU, $	Facility charge, $	Telephone vs video	ROI, $
Current regulation	1.5	65	100	No difference	41 402
Institutional-specific	1.5	65	0	No difference	11 387
Conservative estimate	1.5	50	0	No difference	4633
Medicare fee schedule	1.5	40	0	No difference	131
Break even	1.5	39	0	No difference	0
Potential telephone parity changes	1.5	65	0	Telephone reimbursed at 75% of video	14 414
Potential telephone parity changes	1.5	65	0	Telephone not reimbursed at all	23 496

Abbreviations: ROI, return on investment; RVU, relative value units.

## Discussion

At our institution, the Telehealth Patient Navigator program proved to be an effective, cost-effective, and high-value intervention associated with improving telehealth visit attendance and fewer patient no-shows and cancellations and increased successful video visits over the course of a 12-week pilot. Implementing a Telehealth Patient Navigator may be a high-value proposition for health care systems, as it uniquely benefits patients and clinicians while being cost-effective and yielding a positive net return on investment.

### Limitations

Our study had several limitations. Although patients contacted by the navigator may not have conducted a video visit before, their willingness to answer the phone alone may indicate a favorable view toward use of technology. We used an intention-to-treat principle and considered patients in the intervention group if the navigator attempted to contact the patient. There is risk of selection bias as the navigator called more morning patients than those attending in the afternoon. The combination of a rapid increase in telehealth usage and the relative novelty of video visits likely contributed to the efficacy and value of our navigator program. As the pandemic progresses, it remains to be seen whether telehealth will remain at current usage levels or if patients will return to in-person visits. As patient populations become more familiar with the technology and logistics associated with telehealth visits, the impact of additional technological assistance from a navigator may diminish. If telehealth usage gains traction beyond the pandemic for specific patient populations, such as chronic illness management, the scope of the navigator role can be adapted to support these use cases. Another consideration is that our training standards and workflow were specific to our institution, and may not be generalizable to other institutions with significantly different workflows or patient characteristics. Additionally, we do not yet fully understand the qualifications or qualities that affect a navigator’s effectiveness in their role.

Lastly, the effectiveness of navigator outreach is limited to interactions where the navigator and the patient speak a shared language. The navigator employed for our pilot program was multilingual, speaking French, Haitian Creole, and English, which widened the scope of patients to whom technological assistance could be offered. However, recruiting and retaining a multilingual employee may not always be feasible at other institutions. Our pilot program did not include training on the usage of interpreter services, or on how to perform phone outreach with deaf or hard of hearing individuals via a relay service. As such, the challenges of phone-based outreach to patients requiring an interpreter remains unknown.

Implementing a Telehealth Patient Navigator program and continuing the program comes with some challenges. For the pilot implementation, our program was limited to 1 navigator, and we occasionally experienced time limitations on our ability to reach all of the patients designated for phone outreach who had a next-day appointment. For continuation beyond the pilot, foreseeable challenges include securing up-front robust program funding, delegating management of telehealth navigators (central vs department level), and prioritizing which patients require outreach assuming finite resources for outreach.

Our next steps include refining a prioritization tool to identify patients that would best benefit from the program. Although we are currently focusing on patients without a successful video visit based on medical records, we hope to address those that have had difficulty with video visits despite completion. A flagging system by our physicians would be helpful. In addition, our next priorities include broader research efforts with a focus on equity, community health sites, and specialty care areas. For example, it would be important to identify populations that respond well to patient navigators and devise other mechanisms to address telehealth access in others.

## Conclusions

In our 3-month pilot, we found that a patient navigator was associated with reduced cancellations and no shows, increased video visit uptake, and positive financial value. These efforts may reduce barriers to telehealth and promote equity.

## Notes

Supplement. **eFigure.** Adjusted Odds of Telehealth Video Visit Adherence by Intervention and Demographic Group **eTable.** Telehealth Navigator—Multivariate Logistic Regression Model Results
