# Supplementary material for: Evaluation of Telehealth Visit Attendance After Implementation of a Patient Navigator Program
Source: JAMA Netw Open. 2022 Dec 8;5(12):e2245615. doi: 10.1001/jamanetworkopen.2022.45615 (PMC9856233; doi:10.1001/jamanetworkopen.2022.45615)
Supplement: Supplement. — eFigure. Adjusted Odds of Telehealth Video Visit Adherence by Intervention and Demographic Group eTable. Telehealth Navigator—Multivariate Logistic Regression Model Results [file jamanetwopen-e2245615-s001.pdf]

Supplemental Online Content

Mechanic OJ, Lee EM, Sheehan HM, et al. Evaluation of telehealth visit attendance after implementation of a patient navigator program. *JAMA Netw Open.* 2022;5(12):e2245615. doi:10.1001/jamanetworkopen.2022.45615

eFigure. Adjusted Odds of Telehealth Video Visit Adherence by Intervention and Demographic Group

eTable. Telehealth Navigator—Multivariate Logistic Regression Model Results

This supplemental material has been provided by the authors to give readers additional information about their work.

© 2022 Mechanic OJ et al. *JAMA Network Open.*

eFigure. Adjusted odds of telehealth video visit adherence by intervention and demographic group

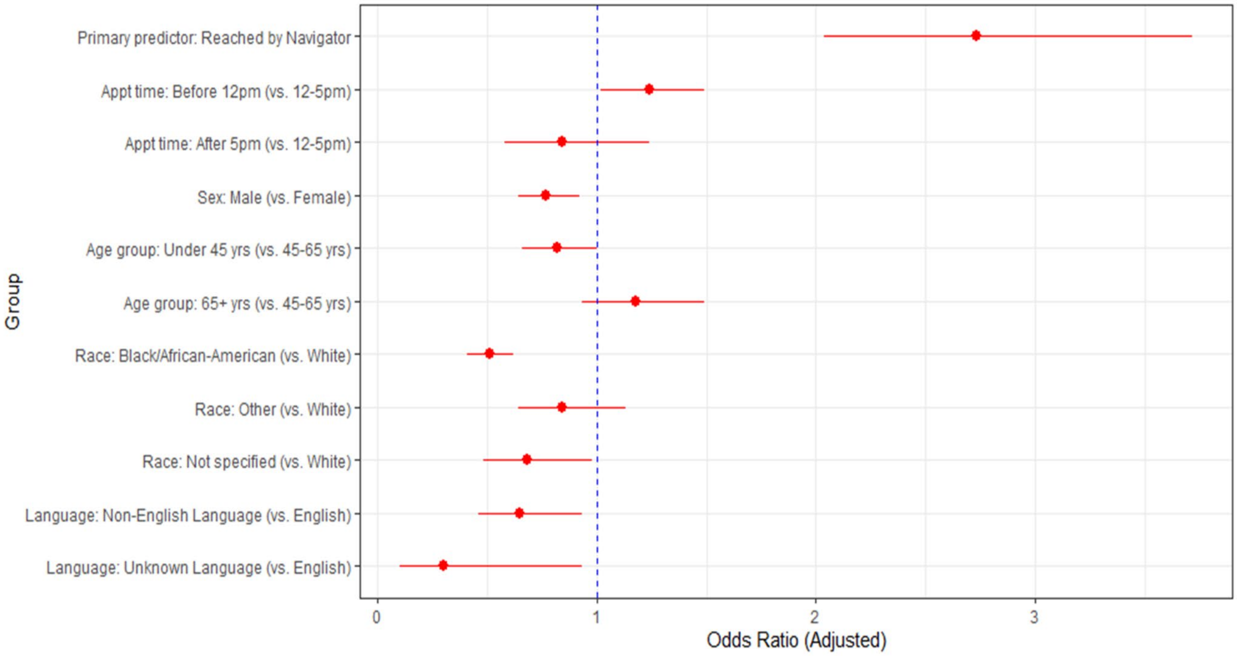

© 2022 Mechanic OJ et al. *JAMA Network Open.*

eTable. Telehealth Navigator – Multivariate Logistic Regression Model results

N = 4,066	
	Odds Ratio<br>(95% Confidence Interval)
**Pilot Arm**	
Intervention (Navigator Outreach)	2.02 (1.59, 2.61)
Comparator (No Navigator Outreach)	Reference
**Appointment Time**	
Before 12pm	1.22 (1.01, 1.48)
12pm to 5pm	Reference
After 5pm	0.84 (0.58, 1.24)
**Age group**	
Under 45 yrs	0.80 (0.65, 0.99)
45 to 65 yrs	Reference
65+ yrs	1.17 (0.92, 1.48)
**Sex**	
Female	Reference
Male	0.76 (0.63, 0.91)
**Race (group)**	
Black/African American	0.50 (0.41, 0.62)
American Indian / Alaska Native / Native Hawaiian / Pacific Islander	0.79 (0.21, 5.15)
Asian	1.27 (0.84, 1.99)
White	Reference
Other[a]	0.61 (0.43, 0.88)
Unspecified[b]	0.69 (0.48, 0.99)
**Language**	
English	Reference
Non-English	0.64 (0.46, 0.92)
Unknown	0.30 (0.10, 0.94)

[a] Race (group): Other denotes Other Race (an option in the electronic health record

[b] Race (group): Unspecified denotes that the data on race was blank, unknown (an option in the electronic health record) or declined to answer

© 2022 Mechanic OJ et al. *JAMA Network Open.*
